# Prevalence and Incidence of Multiple Sclerosis in Tehran, Iran

**Published:** 2017-05

**Authors:** Sharareh ESKANDARIEH, Pouria HEYDARPOUR, Seyedeh-Robab ELHAMI, Mohammad Ali SAHRAIAN

**Affiliations:** 1. Brain and Spinal Cord Injury Research Center, Neuroscience Institute, Tehran University of Medical Sciences, Tehran, Iran; 2. MS Research Center, Neuroscience Institute, Tehran University of Medical Sciences, Tehran, Iran; 3. Dept. of Epidemiology and Biostatistics, School of Public Health, Tehran University of Medical Sciences, Tehran, Iran

**Keywords:** Multiple sclerosis, Prevalence, Incidence, Joinpoint regression

## Abstract

**Background:**

Tehran is the capital of Iran with an increasing Multiple Sclerosis (MS) incidence. A retrospective population-based study was conducted to evaluate the status of MS prevalence and MS incidence trends in Tehran Province, Iran.

**Methods:**

A population-based study was conducted from 1991 to 2014 in Tehran Province, the capital city of Iran based on Iranian MS Society (IMSS) registry system. A structured questionnaire design to cover the important epidemiological variables, related at the individual level for MS. A Monte Carlo Permutation method was utilized to test whether the apparent change in MS trends during 1991–2014 was statistically significant.

**Results:**

MS incidence was significantly increased during our study period. The annual percent change (APC) was 12.8% in women during 1991 to 2004 and 12.5% in men during the same period. The point prevalence of MS was 101.39 per 100000 populations in 2014. The age-adjusted prevalence rates were 134.03 and for male patients were 42.45 per 100000.

**Conclusion:**

MS prevalence and incidence in Tehran are markedly rising, it is crucial to elucidate the causes of the increasing trends and implement efficient policies lowering disease burden.

## Introduction

Multiple sclerosis (MS) is an inflammatory demyelinating disease that affects more than 2.5 million people worldwide (1). Meta-regression analyses of studies on MS epidemiology since 1965 revealed an almost universal increase in prevalence and incidence of MS over time; and suggest a general increase in incidence of MS in females (1). Globally, the median estimated prevalence of MS is 112.0 per 100000 and the median estimated incidence of MS is 5.2 per 100000 (2). Greatest prevalence observed in North America and Europe (140 and 108 per 100000, respectively) (3). Iran is considered as a country with high MS prevalence (51.52 per 100000) in Middle East (4).

The study for prevalence estimation of MS in Iran in 2013 indicated a high prevalence rate in Isfahan (89 per 100000 of population) and Tehran (88/100000), situated at the central part of Iran (5). Numerous studies revealed that having positive family history of MS can play little role in increasing risk of the disease (6, 7). The prevalence and incidence of MS widely vary amongst numerous geographical areas and different countries. The current frequency of MS is still unknown in Tehran.

The aim of this study was to detect significant changes in MS epidemiology in Tehran during 1991 to 2014, calculate the incidence trend, and point prevalence of MS by focusing 2013–2014 in Tehran Province, Iran. We also evaluated the familial MS history and pediatric MS incidence.

## Methods

### Study Area

This population-based study was conducted in Tehran Province, the capital of Iran. Tehran is located in the north/center of Iran with an estimated population of 12559000 in 2014.

### Data source

Iranian MS Society (IMSS) records were studied to obtain annual incidence data (8–10). IMSS is the only center in the surveyed area that registers MS patients and provides wide facilities for the members. Only patients approved by neurologists with MS fulfilling McDonald (11) or the Poser criteria (12) are registered in the IMSS. A trained interviewer explains the purpose of the registry for patients in the IMSS, and after obtaining informed consent, patients are asked to complete structured interview questions. Each person fills out a questionnaire relating to baseline characteristic data such as sex, birth date, familial history of MS and age at disease onset.

The majority of the MS patients are registered in the IMSS, while the number of patients registered during the exact year of disease onset, may remain underestimated.

The Statistical Center of Iran regularly conducts population censuses, including in the year 2013–2014, and estimates the average annual population for the in-between years based on several registries available in the country.

The age-adjusted incidence rates were calculated based on years of disease onset and 2000–2025 WHO standard population. We have also studied the age-adjusted prevalence rate at the end of the study period on Mar 20, 2014.

### Joinpoint regression analysis

Joinpoint regression has been widely used to study various disease trends over time (13–16). Joinpoint software takes trend data and fits the smallest number of change-points supported by the data, that is, where significant shifts of the annual percent change (APC) are observed. These are called “Join points (JPs)”. We found the number of significant JPs in our trend data during 1991–2014 by using JoinPoint Software Ver. 4.0.1.

## Results

### MS incidence trends

Among 15672 MS patients recorded in IMSS in Tehran Province, 749 MS was recorded in 2013 including 565 (75.4) female and 184 (24.6) male and 485 MS recorded in 2014 including 351 (72.4) female and 134 (27.6) male. The MS incidence was 6.02 per 100000 populations in 2013 (9.16 in female and 2.93 in male) and the MS incidence was 3.87 per 100000 population in 2014 (5.63 in female and 2.11 in male) (Table 1).

**Table 1:** Crude incidence and baseline characteristic of MS patients in 2013–2014

**Variables**	**n (%)**	**2013 Population**	**CI**	**n (%)**	**2014 Population**	**CI**
Sex	749			485		
Female (916)	565 (75.4)	6167000	9.16	351 (72.4)	6228000	5.63
Male (318)	184 (24.6)	6266000	2.93	134 (27.6)	6331000	2.11
Age group						
≤19	64 (8.5)	3279000	1.95	36 (7.4)	3286000	1.09
20–24	153 (20.4)	1107000	13.82	89 (18.4)	1030000	8.64
25–29	184 (24.6)	1463000	12.57	131(27.0)	1414000	9.26
30–34	148 (19.8)	1374000	10.77	94 (19.3)	1440000	6.52
35–39	97 (13.0)	1050000	9.23	65 (13.4)	1107000	5.87
40–44	60 (8.0)	908000	6.60	29 (6.0)	917000	3.16
45≥	43 (5.7)	3252000	1.32	41 (8.5)	3365000	1.21

CI: Crude Incidence per 100000 population

**Fig. 1:**
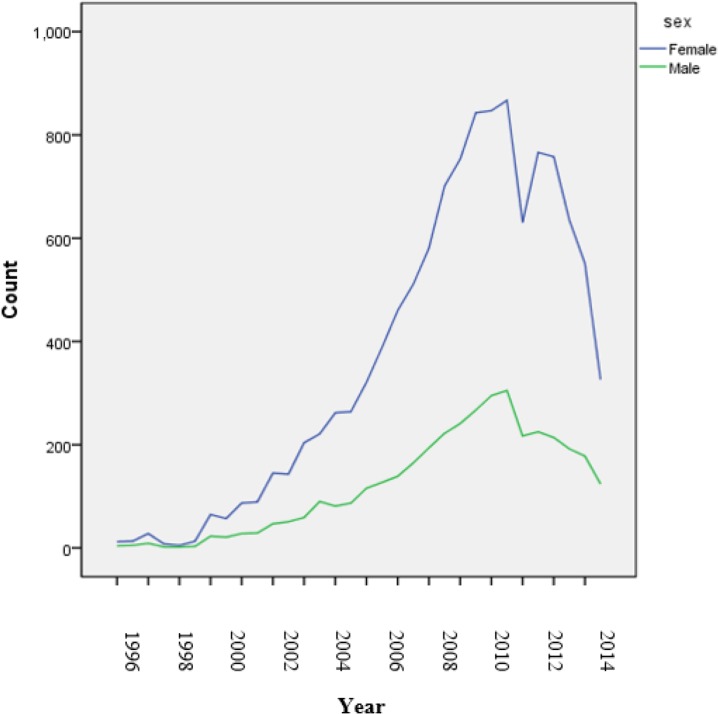
MS incidence via Join point regression analysis in various sex groups

In 2013, most of MS incidence have been seen in patients aging 20–24 yr old, 13.82 per 100000 population followed by 25–29 yr old, 12.57 per 100000 populations as well in 2014. Most MS incidences has been seen in patients aging 25 to 29 yr old, 9.6 per 100000 population followed by 20–24 yr old, 8.64 per 100000 populations (Table 1). During study period from 1991–2014, a significantly increasing trend in MS incidence was observed via Joinpoint regression analysis in various sex groups (Fig. 1). The APC in the first segment (until 2004) of pooled sex groups was 12.9% (10.7%–15.1%, *P*<0.05). The APC in the first segment of female group was 12.8% (10.6%–14.9%, *P*<0.05) and the APC in the first segment of male group was 12.5% (10.2%–14.9%, *P*<0.05). The first JP was observed in 2004 and the second JP was observed in 2008 in various sex groups (Fig. 1).

The average female to male ratio during 1991–2014 was 3.18:1 and in 2013–2014 was 2.88:1 (3.07:1 in 2013 and 2.61:1 in 2014).

As shown in Table 1 patients aging 25–29 yr old at disease onset comprised most of the patients (24.6% in 2013 and 27.0% in 2014) followed by patients aging 20–24 yr old 20.4% in 2013. Mean age at disease onset was 30.04 yr with a minimum and maximum of 8 and 62 yr old, respectively. Mean age at disease onset for female patients was 28.81 yr and for male patients was 29.94 yr with Mode of 26.0 yr.

### MS prevalence

On the prevalence day, 15672 cases with definite MS were identified in Tehran. Total point prevalence of MS was 101.39 per 100000 populations. The age-standardized prevalence rate for female patients was 134.03 and for male patients was 42.45 per 100000.

### MS Incidence by sex and age group

Specific incidence rates in 2013–2014 for female and male in different age groups are presented in Table 2.

**Table 2:** Multiple sclerosis incidence rate in Tehran according to sex and age groups in 2013–2014

**Age group (yr)**	**2013 n (%)**	**Female Total pop**	**Female CI**	**Male n (%)**	**Total pop**	**CI**	**2014 N (%)**	**Female Total pop**	**CI**	**Male n (%)**	**Total pop**	**CI**
≤19	50 (8.8)	1605000	3.11	14 (7.8)	1674000	0.83	27 (7.7)	1607000	1.68	9 (6.7)	1677000	0.53
20–24	118 (20.9)	555000	21.26	35 (19.0)	551000	6.35	64 (18.2)	513000	12.47	25 (18.7)	517000	4.83
25–29	139 (24.7)	738000	18.83	45 (24.3)	725000	6.20	101 (28.7)	713000	14.16	30 (22.4)	701000	4.27
30–34	107 (18.9)	683000	15.66	41 (22.3)	691000	5.93	62 (17.7)	719000	8.62	32 (23.9)	722000	4.43
35–39	70 (12.4)	510000	13.72	27 (14.7)	540000	5.0	48 (13.7)	540000	8.88	17 (12.7)	567000	2.99
40–44	50 (8.8)	443000	11.28	10 (5.4)	465000	2.15	21 (6.0)	445000	4.71	8 (6.0)	472000	1.69
45≥	31 (5.5)	1634000	18.97	12 (6.5)	1619000	7.41	28 (8.0)	1691000	1.65	13 (9.6)	1673000	0.77

Total Pop: Total Population, CI: Crude Incidence per 100000 population

In 2013, MS incidence rate per 100000 populations was 13.8 between 20–24 yr old, 17.30 between 25–29 yr old while in 2014 MS incidence rate per 100000 populations was 25.03 between 20–24 yr old 18.43 between 25–29 yr old in both sexes.

The pediatric MS incidence among patients aging fewer than 18 yr old during our study period was 4.79 among girls and 1.36 among boys per 100000 populations.

### Familial MS history

For the study period from 2013 to 2014, we found 174 (13.97%) patients with a positive family history of MS, 109 (14.6%) among patients during 2013 and 65 (13.40%) during 2014. Concerning patients having history of MS, 14.1% were female and 14.6% were males.

## Discussion

Our study demonstrated a significantly increasing trend in MS incidence in various age groups until 2004. The increasing female to male sex ratio among MS patients during study period from 1991–2014 was observed. The mean age at disease onset among male patients was decreased during the study period, while in female patients the mean age at disease onset was mildly increased.

In Middle East region, prevalence of MS varies considerably across the countries, MS prevalence in Kuwait increased from 4.44 per 100000 in 1983 to 31.15 per 100000 in 2000. Jordan also had a high prevalence (39 per 100000 in 2004–5) of MS, a medium prevalence was observed in Tunisia (9 per 100000 in 1985) and Saudi Arabia had a low prevalence (4 per 100000 in 1989) (17). In Iran, reports from Isfahan indicated that this region has a high prevalence of MS with a recently sharp increase in incidence and prevalence rates (9.1 and 73.3 per 100000, respectively) (18). However, there are reports from other regions in Iran with lower prevalence rates.

The prevalence of MS in north east of Iran ranges between 5.3 to 12.8 per 100000 (19). MS also has a low incidence (2.67 per 100000) and prevalence (13.96 per 100000) in Sistan and Baluchestan, south-east of Iran (20). A Recent study from Iran also reports a prevalence of 88 per 100000 in Tehran Province (5, 8). The MS prevalence at the end of our study period was 101.39 per 100000 inhabitants with 134.03 for women and 42.45 for men.

In our study, a significantly increasing trend was seen in MS incidence until 2004. MS incidence rate was higher in females and patients aging 25–29 yr old. The lowest incidence observed in pediatric groups under 18 yr of age (1).

The decreasing pattern was seen afterward maybe due to time delay between disease onsets and fulfilling the MS diagnosis criteria and delay in registering to IMSS. Several studies have shown an increase in the female to male sex ratio of MS over time (21, 22).

The median estimated male/female ratio is lowest in Europe (0.6), the Eastern Mediterranean (0.55) and the Americas (0.5) and highest in South-East Asia (0.4), Africa (0.33) and the Western Pacific (0.31) (3). The average female to male ratio during our study was 2.88:1. In our study, the modeled male to female sex ratio at birth decreased from 0.48 in 1950 to 0.26 in 1995 (APC of −1.39% with no JPs, *P*<0.05).

The average age of onset is lowest in the Eastern Mediterranean region (26.9) followed by similar average age of onset in Europe (29.2) (3). In our study, the mean age at disease onset was 30.04 yr old and patients aging 25–29 yr old were the most prevalent cases. Although the mean age at disease onset in men (29.94) was higher than women (28.81) in our study, a decreasing pattern was seen in the mean age of onset in male patients while an increasing non-significant trend was seen in the female patients.

In our study, 7.70% of patients studied during 1991 to 2014 experienced their first demyelinating event occurring before 18 yr of age. In major regional MS center in the Northeastern United States, pediatric MS consisted 3.06% of cases (23).

Environmental factors contribute to MS pathogenesis in genetically susceptible individuals. Family history of MS is associated with a significantly increasing of MS risk (24). Our results indicates that, the percentage of patients with positive family history of MS in Tehran Province is higher than other regions of Iran and other countries in middle east like Kuwait and Qatar (9, 25–30).

A positive history of MS increased from 5% in 2003 to 14.55% in 2013 (27). In our study, similar to a study in Isfahan, more men with MS, had family history of MS as compared to women (25). The increasing patterns are seen in MS incidence in Tehran Province over two decades, changes in sex ratio at birth during 1950 to 1995 and increased pediatric MS cases denotes close attention to environmental issues affecting susceptible people.

## Conclusion

Tehran is amongst regions with the highest prevalence of MS and the incidence of MS has been significantly increasing in both sexes during last two decades.

## Ethical considerations

Ethical issues (Including plagiarism, informed consent, misconduct, data fabrication and/or falsification, double publication and/or submission, redundancy, etc.) have been completely observed by the authors.
